# AQuRo: A Cat-like Adaptive Quadruped Robot With Novel Bio-Inspired Capabilities

**DOI:** 10.3389/frobt.2021.562524

**Published:** 2021-04-12

**Authors:** Azhar Aulia Saputra, Naoyuki Takesue, Kazuyoshi Wada, Auke Jan Ijspeert, Naoyuki Kubota

**Affiliations:** ^1^ Graduate School of Systems Design, Tokyo Metropolitan University, Hino-shi, Japan; ^2^ Biorobotics Laboratory, School of Engineering, Institute of Bioengineering, Lausanne, Switzerland

**Keywords:** quadruped robot, bio-inspired model, neural-based locomotion, internal-external sensory information, novel capabilities

## Abstract

There are currently many quadruped robots suited to a wide range of applications, but traversing some terrains, such as vertical ladders, remains an open challenge. There is still a need to develop adaptive robots that can walk and climb efficiently. This paper presents an adaptive quadruped robot that, by mimicking feline structure, supports several novel capabilities. We design a novel paw structure and several point-cloud-based sensory structures incorporating a quad-composite time-of-flight sensor and a dual-laser range finder. The proposed robot is equipped with physical and cognitive capabilities which include: 1) a dynamic-density topological map building with attention model, 2) affordance perception using the topological map, and 3) a neural-based locomotion model. The novel capabilities show strong integration between locomotion and internal–external sensory information, enabling short-term adaptations in response to environmental changes. The robot performed well in several situations: walking on natural terrain, walking with a leg malfunction, avoiding a sudden obstacle, climbing a vertical ladder. Further, we consider current problems and future development.

## Introduction

Robots have become necessary to ease human tasks in many contexts such as industrial, military, entertainment, and disaster settings. Robots have different structures for different purposes. Arm-like robots feature in industrial contexts for performing hand-like functions. Humanoid robots with a wheeled base are often used in social and entertainment contexts. Likewise, robots with legs have an advantage on rough terrain, making them suitable for military and disaster contexts. From a broader perspective, legged robots are more versatile than wheeled robots simply because less than half of the world’s terrain can be accessed on wheels.

There are currently many varieties of legged robot exhibiting inspired designs and performance. Boston Dynamics has built many quadruped robots that have excellent capability on rough terrain (Ackerman, 2016). Quadruped robots developed at Waseda University have also demonstrated performance on rough terrain and in ladder-climbing (Hashimoto et al., 2019). Their movement, however, seems slow compared with existing quadruped robots. Most legged robot researchers implement biological structures of quadruped animals to benefit from the animal’s performance. MIT, for example, has built a Cheetah-like robot that moves at high speed (Hyun et al., 2014). BigDog (Raibert et al., 2008), Spotmini (Ackerman, 2016), HyQ (Semini et al., 2011), and Laikago (Spectrum, 2019) are inspired by dogs. They show flexibility of omni-directional movement on natural terrain. Ijspeert’s group took their inspiration from salamanders (Crespi et al., 2013). Animal-inspired robots, however, draw their mobility capabilities from the animals that they are designed after. In contrast to dogs and salamanders, cats are able to climb as well as walk, run and leap over rough terrain. Their claws allow agile climbing behaviors. We have therefore proposed a quadruped robot inspired by feline morphology. We propose a unique paw structure with a gripping mechanism.

The proposed robot is equipped with physical and cognitive capabilities, which include: 1) affordance perception for movement behavior, 2) path planning, 3) a dynamic locomotion generator, 4) stabilization behavior.

For the movement-related perception process, researchers have used different sensors and different strategies. LittleDog (Kolter et al., 2009) used stereo-vision to build the terrain model for the space in front of the robot. Then, it performs footstep planning for the next stepping movement (Kolter et al., 2008). Other researchers have done similar work in perception strategy (Diebel et al., 2004)(Gao et al., 2007). Havoutis *et al.* used an RGBD camera to perceive environmental conditions. Their robot then generates a motion pattern and undertakes foothold planning (Havoutis et al., 2013). Their subsequent work continues on to advanced implementation, such as stair-climbing (Winkler et al., 2015). The MIT Cheetah robot performs impressively while running and jumping to avoid an obstacle (Park et al., 2015). This robot uses LRF (laser range finder) sensors to detect upcoming obstacles, and identifies them using an iterative end-point fitting (IEPF) algorithm. Once an obstacle is perceived, the robot prepares the jump by controlling speed.

Manchester *et al.*, used more complex external sensors such as vision, laser, and radar sensors. Their robot builds a terrain map model and then generates a sequence of footstep locations and associated joint trajectories. The perception is only effective on slow timescales. The footstep planning is updated in every footstep (Manchester et al., 2011). The high-rate timescale is used only for internal sensory response. Many researchers also conducted footstep planning, updated at every footstep, in both bipedal (Deits and Tedrake, 2014) (Maier et al., 2013) (Kuindersma et al., 2016) and hexapodal robots (Belter and Skrzypczyński, 2011). Taking a different approach, Hoffmann *et al.* use a closed-loop strategy for perception and action. They developed interaction between the robot’s embodiment and its environmental context. The robot adjusts its gait or speed when environmental changes are detected (Hoffmann et al., 2011). In this work, the robot reconstructs its map before generating motion plans that address only high-level motion (speed, step length, step height). Next, the stability model controls the low-level motion. The external sensory information is hence not directly used in low-level motion planning. In our proposal, the cognitive model plays a role in the lower-level locomotion model. Using external sensory information and a laser sensor costs less in computational processing to detect object shapes, than using a vision sensor.

The locomotion generator, as its name suggests, generates the movement behavior appropriate to particular conditions. There are many models for legged-robot locomotion. Most researchers implement trajectory-based locomotion for its simplicity; this has been done in bipedal (Manchester et al., 2011)(Zhang et al., 2014b)(Nandi et al., 2016)(Saputra et al., 2015a)(Khusainov et al., 2016)(Saputra et al., 2015c), quadrupedal (Winkler et al., 2015)(Kolter et al., 2008)(Mastalli et al., 2017)(Zhang et al., 2016)(Matsuzawa et al., 2016), and hexapodal robots (Qian and Goldman, 2015) (Zhu et al., 2016). Trajectory-based models control the motion planning in Cartesian coordinates using polynomial equations or Bézier curves (Manchester et al., 2011). Other researchers use center-of-gravity–based trajectory generation for quadrupedal robots (Winkler et al., 2015)(Mastalli et al., 2017). These center-of-gravity trajectory models have been successfully implemented for complex terrain. However, this approach has proven lacking on dynamic locomotion behavior. The trajectory-based approach needs to plan scenario motion planning in advance, and requires extensive parameter-tuning.

On the other hand, some researchers have tried other ways to develop dynamic locomotion patterns that can synchronize automatically with sensory feedback. They consider natural processes to develop locomotion models from human and animal gaits. Quadrupedal animals can generate gait patterns (walk, pace, amble, trot, gallop) automatically, depending on the animal’s intentions and environmental conditions. The animal’s body structure also regulates the gait pattern, which means every kind of animal has different gait efficiencies. Nakada *et al.* propose a neuromorphic locomotion model with a CMOS (Complementary Metal Oxide Semiconductor) controller for inter-limb coordination in quadrupedal robots (Nakada et al., 2003), while other researchers propose central pattern generation (CPG) for quadrupedal robot locomotion (Ijspeert and Cabelguen, 2006)(Asadi et al., 2015)(Maufroy et al., 2008)(Zhang et al., 2014a)(Sun et al., 2018)(Liu et al., 2018). Ijspeert’s group proposed CPG–based control of their salamander robot (Ijspeert and Cabelguen, 2006), which can transition dynamically from walking to swimming. Transitional movements in quadruped robot have also been proposed using CPG model by several researchers (Maufroy et al., 2010; Fukuoka et al., 2015; Owaki and Ishiguro, 2017). Other researchers have developed integration between CPG and ground reaction feedback to synchronize the gait with terrain conditions (Maufroy et al., 2008). Zhang *et al.*, for example, designed a CPG-based controller for trotting (Zhang et al., 2014a). CPG gait generators can be implemented using a spiking neural network (Espinal et al., 2016) or a recurrent neural network (Tran et al., 2014). Sun *et al.* used a decoupled neural CPG circuit for adaptive locomotion (Sun et al., 2018). In our previous model, we combined the CPG with a Bézier curve model for efficiency. We implemented our ideas in a small quadrupedal robot, but it showed limitations on handling variant gait (Saputra et al., 2016)(Saputra et al., 2015b). The quadrupedal robot proposed in the present paper will be implemented as an efficient neural-based locomotion model using a single-rhythm generator-based CPG model, and will include a reflex system for synchronizing with locomotion events. Here, the reflex system is composed as the muscle reflex system explained in (Saputra et al., 2020b) and sensory afferent from force sensor in each leg explained in *Affordance Detection for Grasping*.

Our robot is equipped with external and internal sensors. We use point-cloud data information generated by a laser depth sensor as external sensory information. There are many robots that effectively detect and recognize obstacles using depth sensors (Park et al., 2015)(Hashimoto et al., 2019)(Camurri et al., 2015). The WAREC robot, for example, has a rotating laser range-finder array for scanning the surrounding environment (Hashimoto et al., 2019). Since depth sensors are limited in frequency rate, size, weight, and range, we propose a light-weight array of time-of-flight sensors which alleviates these limitations. To provide internal sensory information, we use an inertial measuring unit (IMU), four force sensors, and four grip-touch sensors.

This paper is organized as follows: *In Design of Robot’s Hardware*, we describe the robot’s mechanical and hardware design. *Movement-Related Capabilities* examines the robot’s unique capabilities. *Robot Implementation*tn shows the implementation of the robot and demonstrates its effectiveness. Finally, in *Conclusions and Future Plans*, we conclude the paper.

## Design of Robot’s Hardware

**FIGURE 1:**
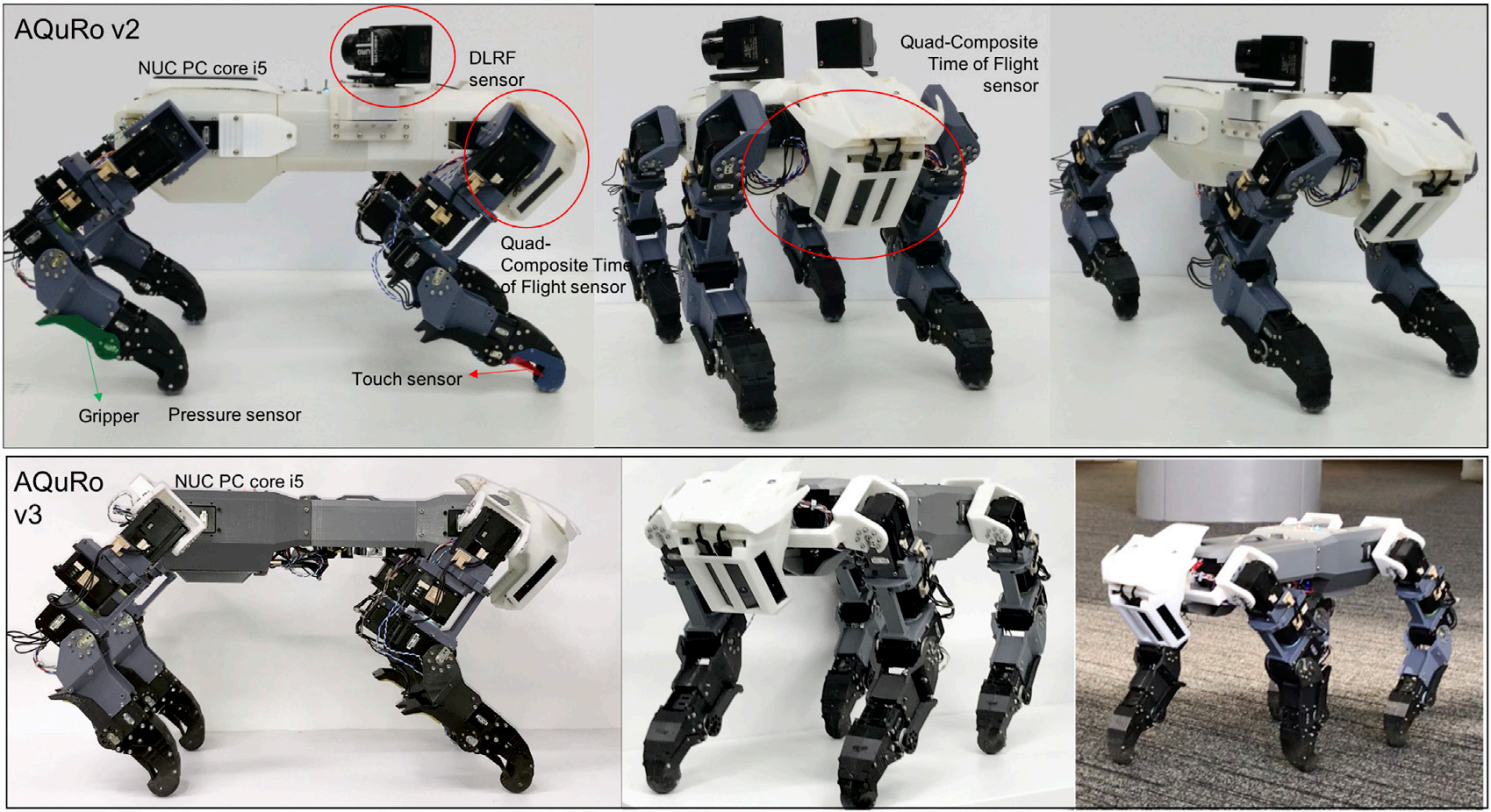
The quadruped robot. AQuRo v2 is attached with DLRF sensor and AQuRo v3 has slimmer body without DLRF sensor.

As stated in the Council on Competitiveness—Nippon (COCN) report, robots suitable for use in disaster situations must be able to move over all of rough, sloped and natural terrain (grass, uneven soil), through narrow spaces, and be able to climb stairs and vertical ladders (Council on Competitiveness Nippon (COCN), 2013). When we seek inspiration from the animal kingdom, the cat family (Felidae) stands out as able do all of these things. Cats can handle many complex environmental conditions. They can swim, are agile, and can climb trees. The cat offers a most appropriate archetype to imitate in agile quadrupedal robots. The feline robot that we developed is shown in Figure 1.

### Mechanical Design

**FIGURE 2:**
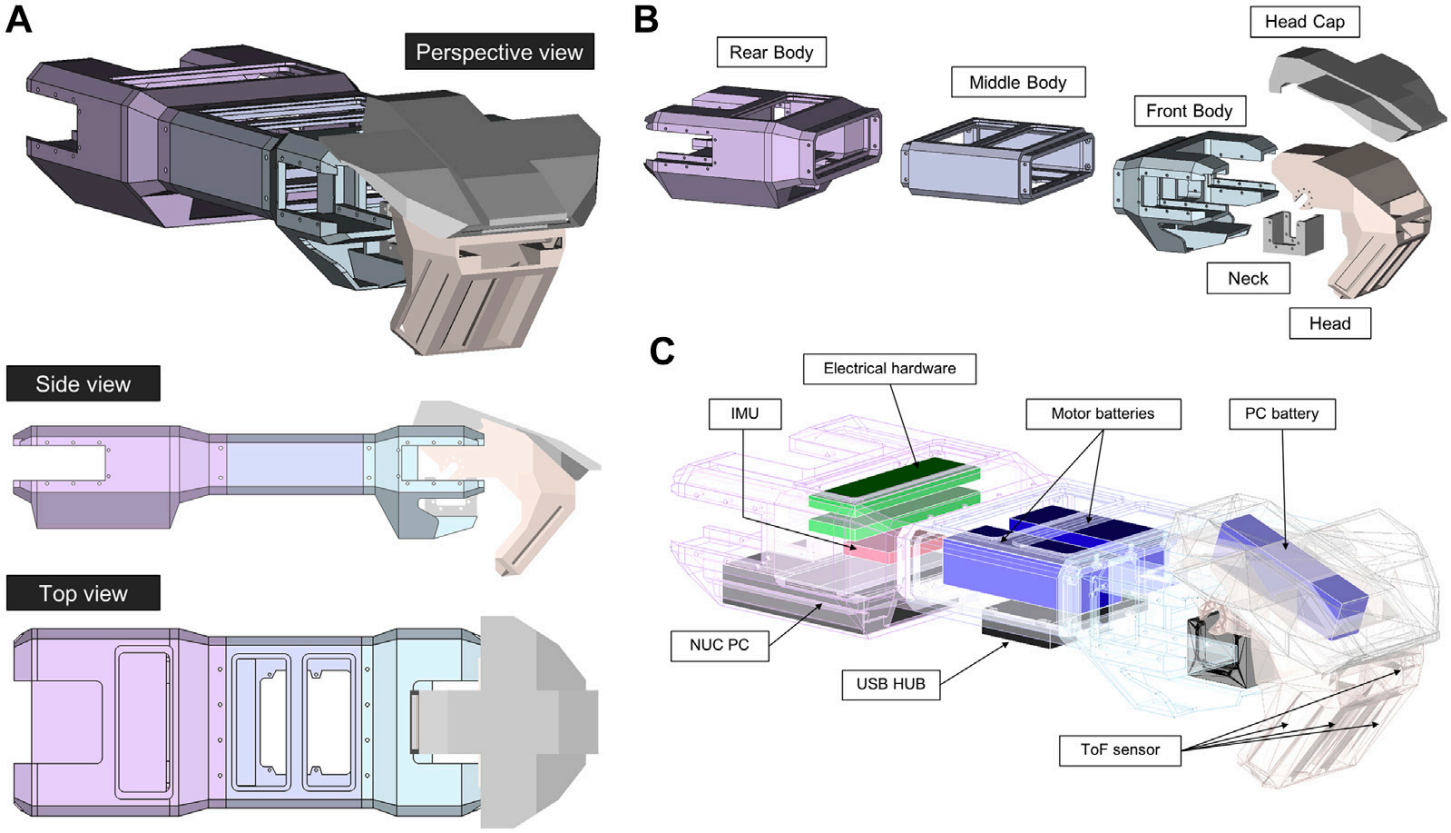
The robot body (torso and head). **(A)** Orthographic projections. **(B)** Interior hardware placements. **(C)** Exploded parts.

**FIGURE 3:**
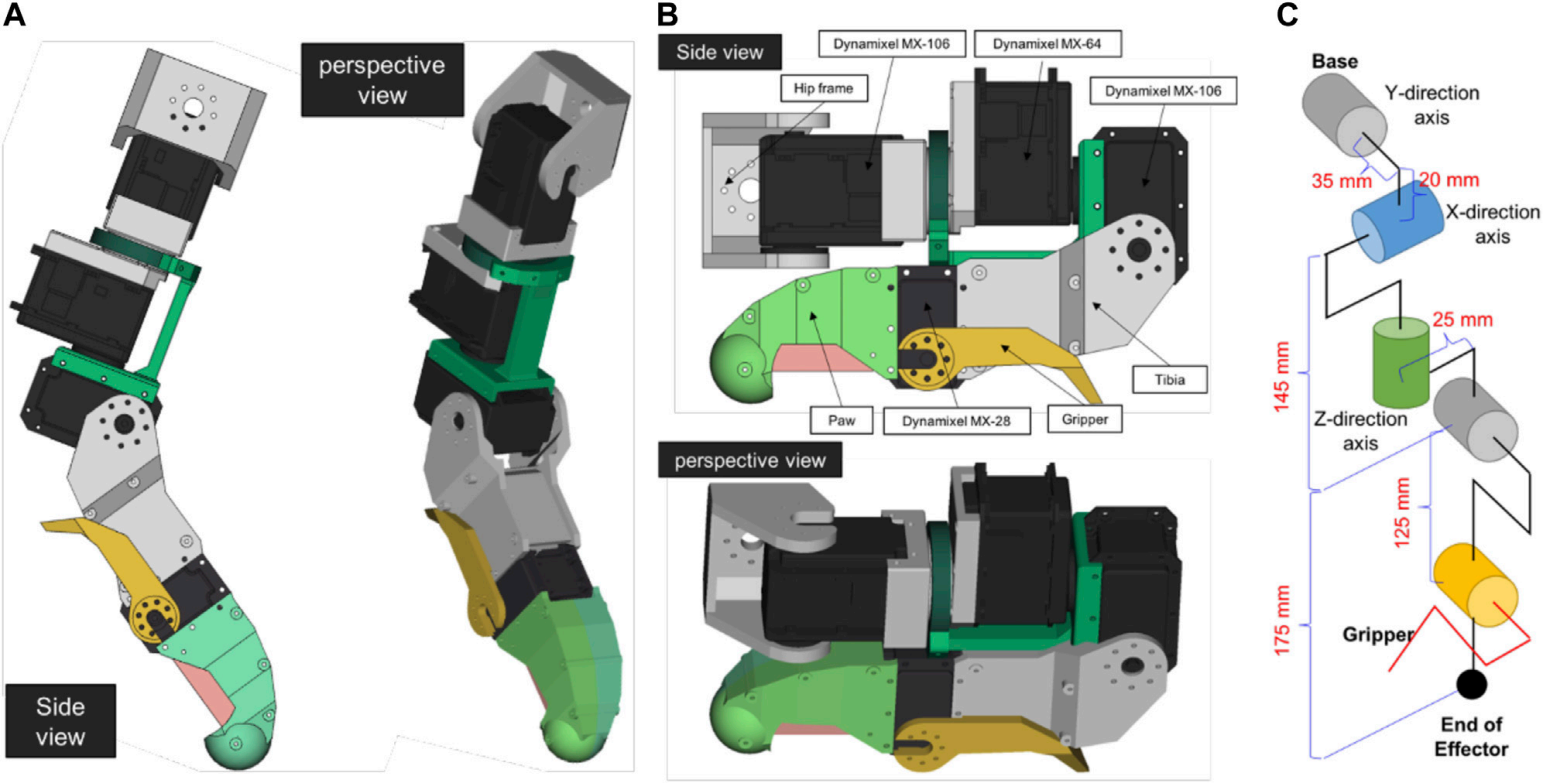
The design of Leg **(A)** flexed, **(B)** extended. **(C)** Leg actualization.

Our proposed quadrupedal robot is similar in size to a mature domestic cat: 25 cm (width) × 60 cm (length) × 30 cm (height). The robot has around 7 kg of weight. Figure 2 The robot’s foreleg imitates the cat’s forelimb structure minus the wrist joint. It has only two joints, the shoulder and elbow. There are three actuators associated with the ball joint structure of the shoulder, and one actuator associated with the hinge joint structure of the elbow. To design the robot’s hindleg, we considered the cat’s rhythmic motion, in which the proximal and distal leg segments maintain their relative angular orientation during most of the cycle, the deviation of angular joints differing only at the onset of toe-off (Witte et al., 2001). In the hindlegs, therefore, we simplified by eliminating the knee joint so the ankle and hip joints could be directly integrated. The leg can be seen in Figure 3. There are five degrees of freedom in each leg, one of which is used as the gripper joint. The tibia is 175 mm long, and the femur is 145 mm long. The robot’s Denavit-Hartenberg parameters are summarized in Table 1.

**TABLE 1:** DH Table of the joint leg structure.

Joint	Hip-*y* (mm)	Hip-*x*	Hip-*z* (mm)	Knee
α_*i*_	*π*/2	*−π*/2	*−π*/2	0
*a*_i_	20	0	25	172 mm
*d*_i_	35	0	145	0
*θ*_0*,j*_	*π*/2	*−π*/2	*−π*/2	*−π*/2
*θ*_*i*_	*θ*_*0,1*_ + *θ*_1_	*θ*_*0,1*_ + *θ*_2_	*θ*_*0,1*_ + *θ*_3_	*θ*_*0,1*_ + *θ*_4_

#### Robot Body

The robot’s overall body shape can be seen in Figure 1. The shell was 3D-printed in poly-(lactic acid) (PLA). The robot body comprises three parts: rear, middle, and front. The rear legs are attached to the rear part, which also holds the NUC PC, IMU sensor, and electrical hardware. The middle part holds two batteries for the motors and a USB Hub. The front part provides an attachment point for the neck and head. The head holds a battery for the PC.

#### End Effector

We designed the end effector to support agile movements such as walking on rough terrain and climbing vertical ladders. The end effectors must also measure the ground reaction force, and must satisfy size constraints. When climbing, cats use claws to grasp rocky walls, trees, poles, etc. Shiquan *et al.* developed an end effector with a dense array of micro-spines (Wang et al., 2016) for rock climbing. It needs a larger space, however, than is appropriate for our proposed robot. Furthermore, cats grasp by using two limbs in concert. Cats also find it difficult to climb vertical ladders.

**FIGURE 4:**
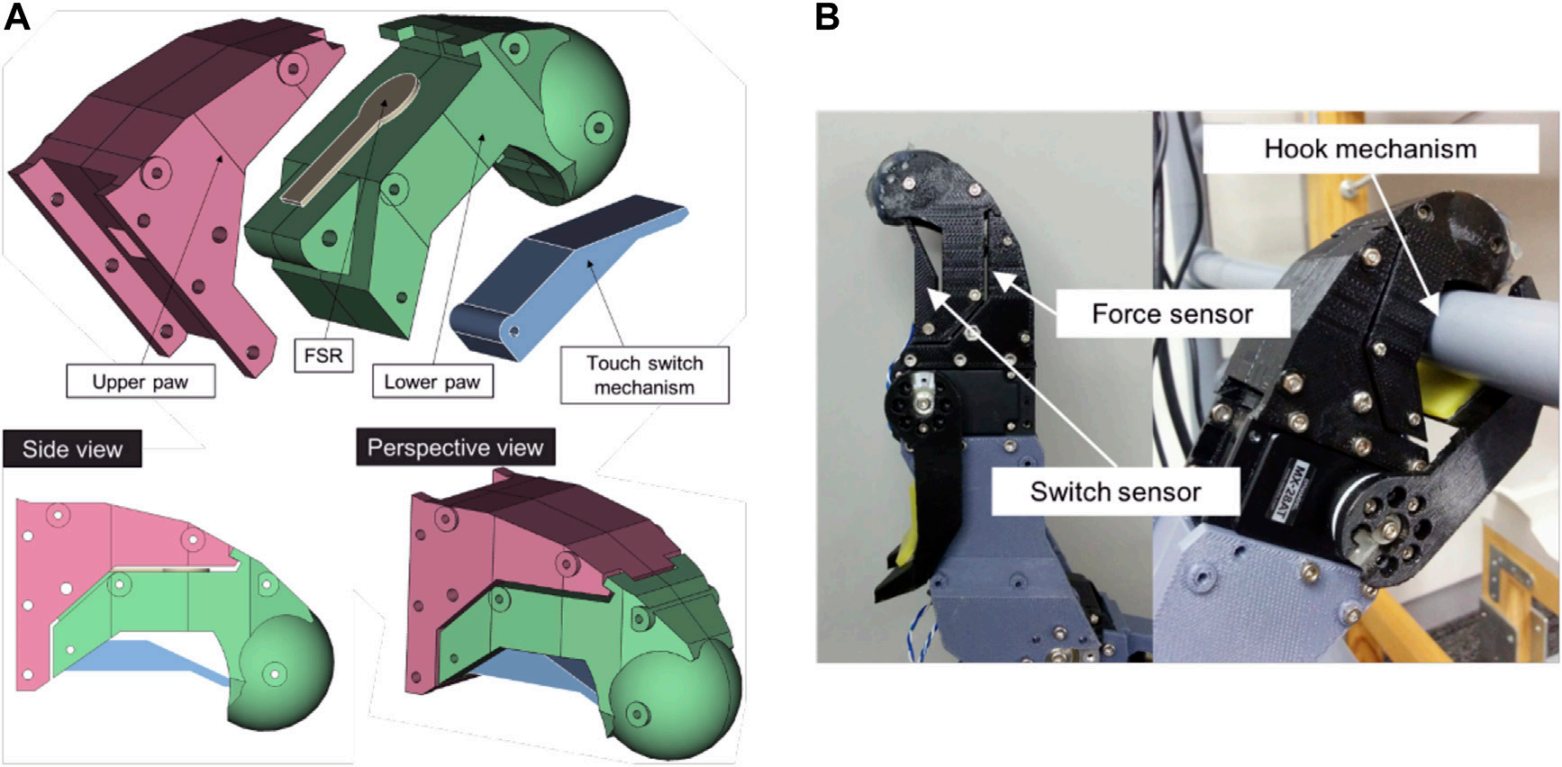
The end effector. **(A)** CAD drawings **(B)** The 3D-printed effector.

In contrast, humans and monkeys have hands to hold and hang from ladder rungs. However, the hand mechanism for such hanging behavior needs a huge torque, which would require a correspondingly bigger servomotor. We simplified using a hook-shaped end effector that requires no actuator. The design can be seen in Figure 4. The end is rounded to simplify footing, eliminating the need for an actuator. Furthermore, in the sensory design, we put a force-sensitive resistor (FSR) between the upper and lower parts of the paw. A switch inside the hook cavity serves as a sensor to detect whether the paw is hooked over a rung. Behind the paw is a moveable claw for grasping and for supporting the hindleg to stand on a rung. The claw, moved by a low-torque servomotor, helps to avoid slippage.

### Sensory System

We provided the cat robot with several sensors representing exteroceptors and interoceptors. To represent the exteroceptors, we built a quad-composite time-of-flight sensor for detecting the surroundings in front the robot, and a dual-laser range-finder for observing more widely. To represent interoceptors, we installed force sensors (force-sensitive resistors) and touch sensors (microswitches) in each leg, and an inertial measurement unit (IMU) inside the body. We use IMU module NG-IMU, as specified in Table 2.

**TABLE 2:** NG-IMU sensor specifications.

Model	NG-IMU
Sensors	Gyroscope, Acceleromoter, Magnetometer, Pressure, Humidity
Update Rate	400 Hz
Static Accuracy (pitch/Roll)	<1 [deg] RMS
Static Accuracy (Heading)	<2 [deg] RMS
Communication	USB, Serial, WiFi
Size and weight	50 × 33 × 8 [mm], 10 [gram]

#### Quad-Composite Time of Flight Sensor

**FIGURE 5:**
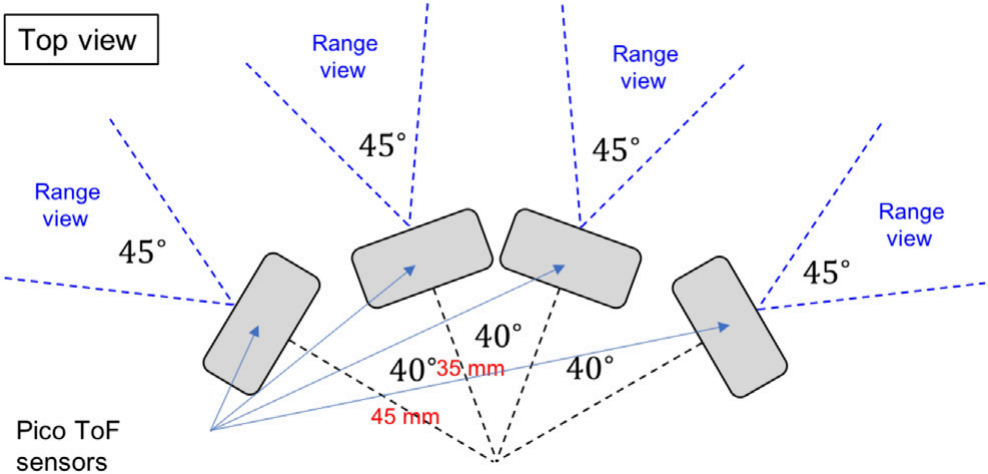
The quad-composite time-of-flight sensor arrangement.

**FIGURE 6:**
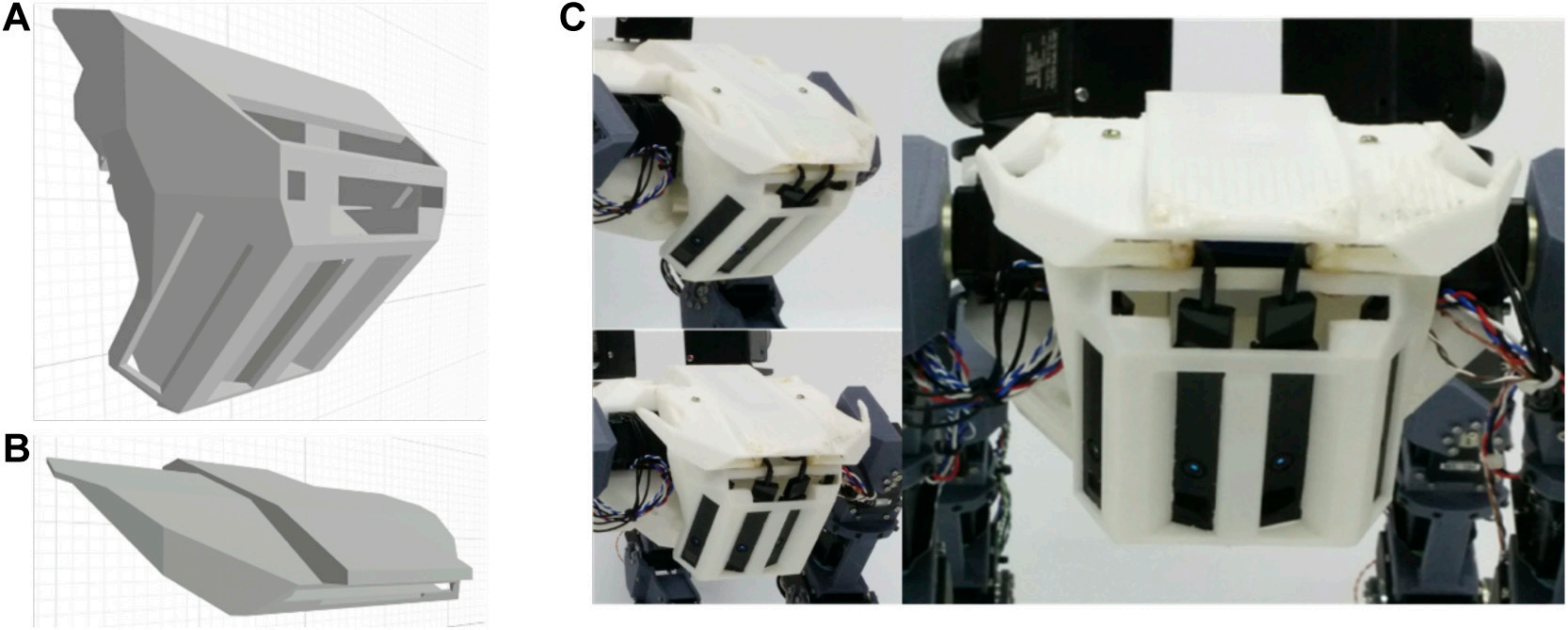
The quad-composite time-of-flight sensors. **(A)** Head housing; the sensors are mounted inside the downward-facing slits. **(B)** Head cap. **(C)** The head with installed sensors.

This sensor will be installed in the head of the robot. It combines four CamBoard pico flexx ToF sensors made by pmd, as specified in Table 3. The composite sensor structure is depicted in Figure 5. This design, combined with the robot’s head shape, provides a wide field of view in order to minimize the number of actuators needed in the robot’s neck. The neck hence contains only one actuator, rotating in the sagittal plane. The CAD design drawings and photographs can be seen in Figure 6.

**TABLE 3:** Time-of-flight sensor specifications.

Model	CamBoard pico flexx
Dimension, weight	68 × 17 × 7.35 mm, 8 g
Measurement range	0.1–4 m
Framerate	Up to 45 fps (3D frames)
Resolution	224 × 171 (38k) pixels
Viewing angle (H x V)	62 ° × 45 °

#### Dual-Laser Range Finder Sensor (DLRF)

**FIGURE 7:**
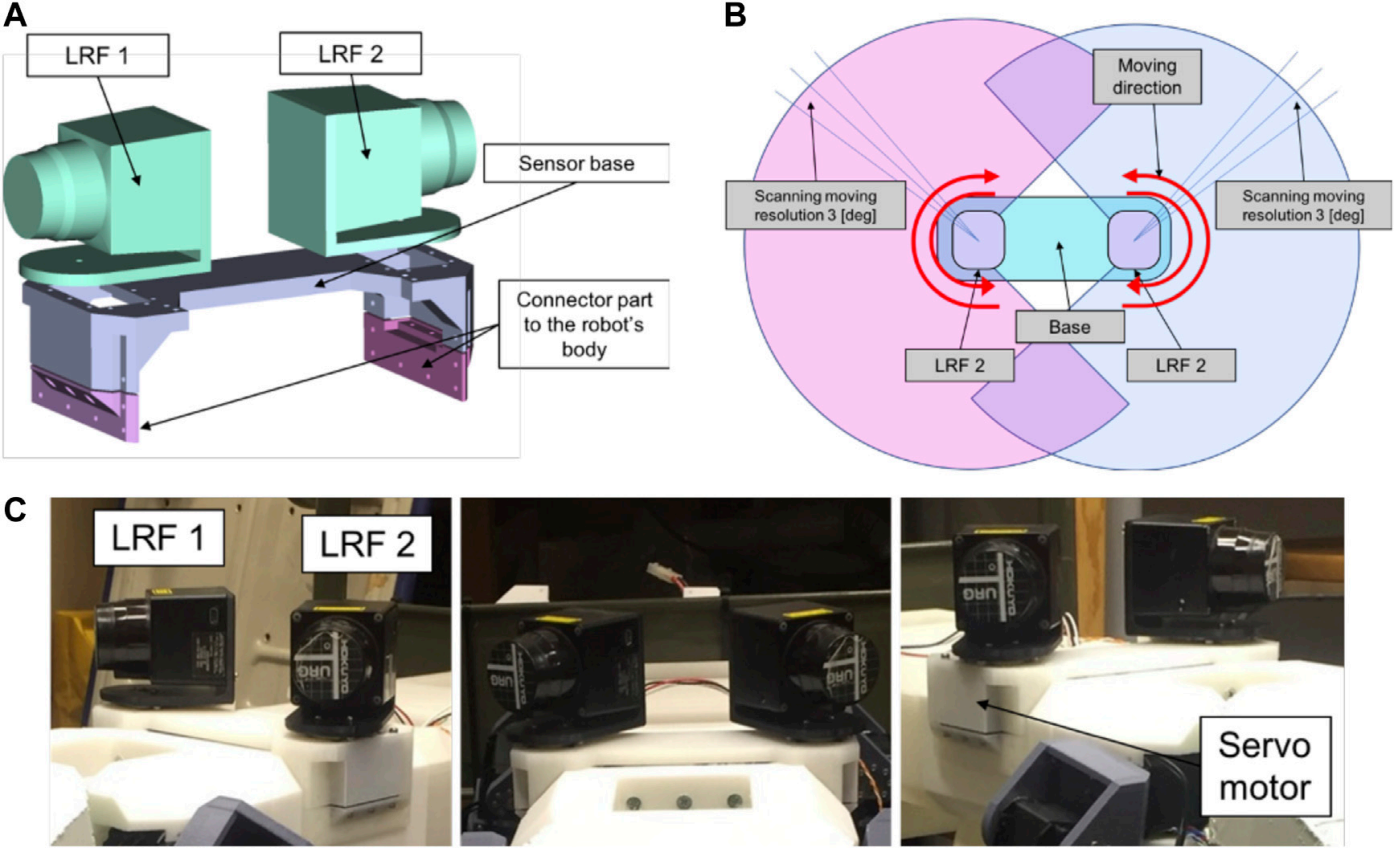
Design of DLRF sensor. **(A)** CAD design. **(B)** The structure of the sensor **(C)** The appearance design of the Dual LRF sensor.

The DLRF is composed of two LRF sensors, with each LRF is attached to a Dynamixel MX-28 servomotor. This mechanism allows the sensors to measure distances. The design can be seen in Figure 7. Table 4 shows the specifications of the LRF sensor used. In Figure 7C we can see the moving mechanism of the sensors. The sensors will move symmetrically, where if the left sensor moving clockwise then the opposite sensor will move counterclockwise. Each sensor will move 240 [degree] of range. After reaching the limit degree, then the sensor will move the opposite direction.

**TABLE 4:** LRF sensor specifications.

Model	URG–04LX–UG01
Weight	160 g
Measurement range	20–5600 mm
Scanning time	100 ms
Scanning accuracy	60–1000 mm: ±30 mm; 1000–4095 mm: ±3 mm
Measurement range	240°

#### Electrical Hardware

**FIGURE 8:**
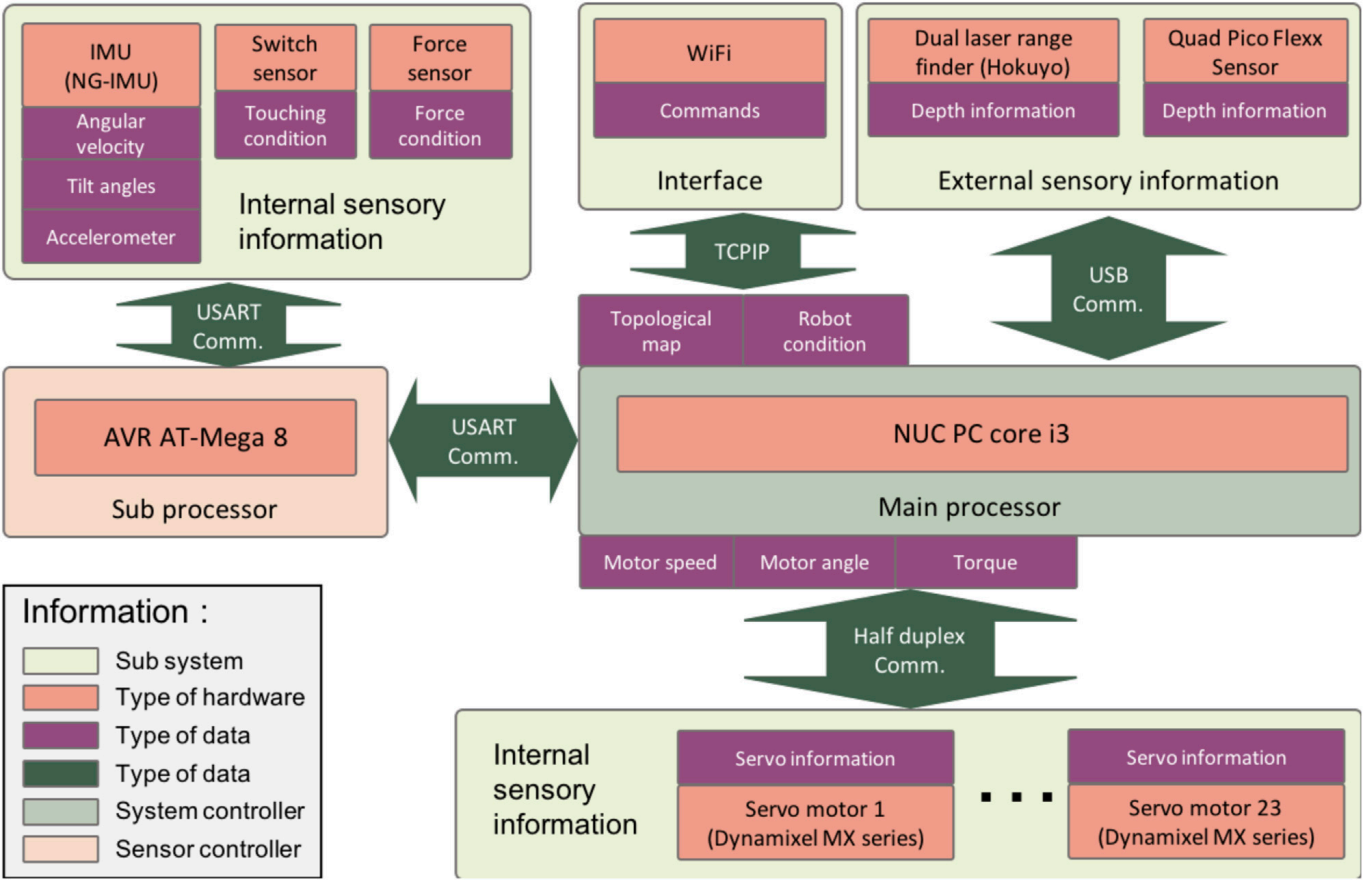
Structure of the electrical system.

The robot has been equipped with a hardware configuration to handle both the internal and the external sensory information. The hardware structure can be seen in Figure 8. We use an ATmega 8 microcontroller as the sub-controller for pre-processing the internal sensory inputs from the force sensor, touch sensor, and IMU. A NUC PC core i3 serves as the main controller for processing several advanced systems such as perception, motion control, communication, and interfacing.

The sub-controller processes analog signals from the force sensor, touch sensor, and IMU sensor through its analog to digital converter input. The data stream is then transferred to the main controller via a USB connection. All external sensory information is also conveyed via USB connections. The main controller generates digital motor control signals for all of the servomotors. There are 12 servo motors Dynamixel MX-106, four servo motors Dynamixel MX-64, and seven servo motor Dynamixel MX-28.

The electrical system is powered by two 4–cell lithium–polymer batteries holding 2700 mAh (14.8 V) and one 4–cell lithium–polymer battery holding 2200 mAh (14.8 V). The batteries are expected to be power the robot for around 15 min.

## Movement-Related Capabilities

We implemented the robot’s movement-related capabilities by integrating external and internal sensory information. This integration allows external sensory information to inform movement behaviors in short adaptation times, as happens in animals: when an obstacle suddenly appears during the walking swing phase, the swing is changed in response, bringing the foot into a safe area. This mechanism illustrates the importance of external sensory information (in this case, vision) for movement behavior.

To use depth information or a 3D point-cloud data as the external sensory information, our robot’s processing system includes 1) a dynamic-density topological map-builder with an attention model, 2) an affordance perceptor using the topological map, and 3) a neural network-based locomotion model.

### Dynamic-Density Topological Map-Building With Attention Model

We present a novel algorithm to realize an attention mechanism for robot movement, based on the dynamic density of a growing neural gas. The aim of this model is to reduce the data representation overhead associated with the 3D point-cloud data. The basic real-time Growing Neural Gas (GNG) technique has been implemented in our previous path planning model (Saputra et al., 2017). We extended the GNG by adding a dynamic-density model. The algorithm’s details are given in (Saputra et al., 2019b)(Saputra et al., 2019a). A comparison between the common GNG and the proposed GNG augmented with dynamic attention can be seen in the link of Video 1.

### Affordance Perception Model

The concept of affordance originated from Gibson, in ecological psychology (Gibson, 1977). Turvey describes affordance as the environment’s dispositional properties. The actor’s effectivity or dispositional properties will supplement what the environment provides. Affordance provides important details governing the actor’s potential behavior and capability. A difference in the robot’s embodiment can therefore lead to different affordance perceptions (Turvey, 1992). The aim of our proposed affordance perception model is to find a suitable integration between environmental conditions and possible actions for the robot. We built affordance perception systems for the robot’s locomotion, ladder detection, and grasping.

#### Affordance Detection for Locomotion

**FIGURE 9:**
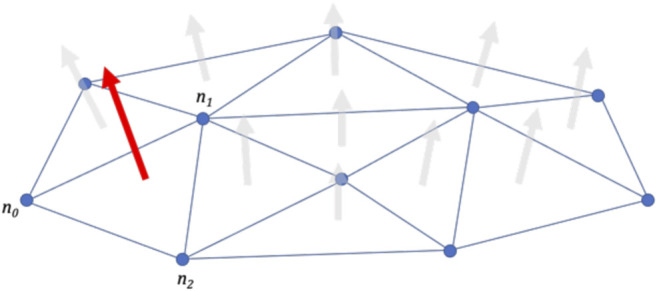
Calculating the normal vector of a plane triangle.

In the locomotion system, the active behavior is regulated by perceiving the affordances. Prospective actions are therefore produced according to the affordance information obtained. In our model, affordances are detected by examining planes in the topological map generated by the dynamic-density growing neural gas (DD-GNG). The affordances of interest are horizontal (or nearly horizontal) surfaces that the robot can step on. These are found by calculating the plane’s slope. We calculated the normal vector of triangular facets in the topological structure using Eq. 1, as illustrated in Figure 9.

$$N=\frac{\left(n_{0}-n_{1}\right)\times \left(n_{0}-n_{2}\right)}{\left\|\left(n_{0}-n_{1}\right)\times \left(n_{0}-n_{2}\right)\right\|}$$ (1)

After that, the slope of plane facet (*γ*_*i*_) in every surrounding surface needs to be calculated using Eq. 2. A safe-to-step-on factor can then be calculated by considering how vertical the plane is.

$$\gamma_{i}=\cos^{-1}\left(\frac{{\vec{\mathrm{Ob}}}_{i}^{\left(v\right)}\Delta \;{\vec{N}}_{\left(\mathrm{ver}\right)}}{{\vec{\left\|Ob_{i}\right\|}}^{\left(v\right)}\Delta \;\left\|{\vec{N}}_{\left(\mathrm{ver}\right)}\right\|}\right)$$ (2)

Where, ${\vec{Ob}}_{i}^{\left(v\right)}$ is the normal vector of the *i*th obstacle plane and ${\vec{N}}_{\left(ver\right)}$ is the vertical unit reference vector, [010].

#### Affordance Detection for Vertical Ladders

The model aims to present low-cost real-time vertical ladder-detection from 3D point-cloud data. The output from the DD–GNG is used as input to the affordance model. Feature-extraction is needed to identify suspected artifacts for the next stage of processing. Thereafter, vertical ladder-rung detection is processed using an inlier–outlier system. The ladder detection system thereby represents the ladder as a set of nodes and edges. Next, we detect the graspable locations by considering the robot’s embodiment. The details of the proposed detection system can be seen in (Saputra et al., 2019a).

#### Affordance Detection for Grasping

This affordance detection process aims to detect possible gripping positions on the object. The process generates a seven-dimensional representation of grippable locations: (3D location, 3D rotation, and object diameter). We put an RGB camera above the robot’s quad-composite ToF sensor to detect the target object. Detection of target objects is performed by a computer vision algorithm. After that, the topological structure will be generated by using the proposed DD–GNG. The density of the topological structure is centralized on the desired object. Based on the inlier–outlier process, the possible gripping information is determined from topological map information and the robot gripper embodiment. Gripping possibilities can then be ranked from ‘best’ to ‘worst’ in any identified gripping solution. Details of this process can be seen in previous research (Saputra et al., 2019b).

### Locomotion Model

Our locomotion model responds to current CPG development challenges in quadruped locomotion research. We present an efficient and solid CPG model that dynamically integrates with sensory feedback for generating various gaits, and allows for leg malfunction compensation without greatly increasing the number of parameters involved. The model has two feedback mechanisms based on sensorimotor coordination (Rossignol and Frigon, 2011)(Lam and Pearson, 2002). In the first feedback mechanism, sensory feedback is used to adjust CPG modulation. This is done by feeding proprioceptive signals representing the leg’s force exertion and swing phase back to the rhythm generator neurons (RG). This feedback is reduced by the second feedback mechanism, when legs are injured. A nociceptor neuron in the injured leg sends a signal to modify the effects of that same leg’s other sensory signals to the RG. Furthermore, we integrate the locomotion functions with supraspinal-level functions generated from cognitive information. Our overall model mimics the descent of influence from attention mechanisms driven by visual information down to muscle activation. Our model addresses the problem of providing short-term adaptation in response to perceiving a sudden obstacle Table 5.

**TABLE 5:** Table of parameters.

*v*_*i*_	Inhibition effect of its self-adaptation
y_*j*_	Signal from other *j*-RG neurons, *y*_*j*_ calculated as *y*_*j*_ = max (*x*_*j*_,0)
***w***_(*RG*,*ij*)_	Synaptic weight of *j*-RG neuron and *i-*RG neuron
τ and *T*	The inner-state and self-adaptation effects
α_*i*_	sensory feedback of *i*-RG neuron
α_*i,0*_	Basic stimulation of the *i*th neuron
***w***_(*FR*,*ij*)_ and ***w***_(*SR*,*ij*)_	The synaptic weights of the force afferent (*F*_*i*_) and the swing-phase afferent (*S*_*i*_) of the *i*th leg to the *j*th RG neuron
***w***_(*NS*,*ij*)_	The synaptic weights of the nociceptor afferent (*N*_*i*_), a pain receptor that detects the condition of leg damage and sends damage stimuli to RG neurons
*G*_*STIM*_	The gain parameter controlling the relationship between speed stimulation *S*_*STIM*_ and the sensory network
*τ*_*f*_	Frequency control parameter

**FIGURE 10:**
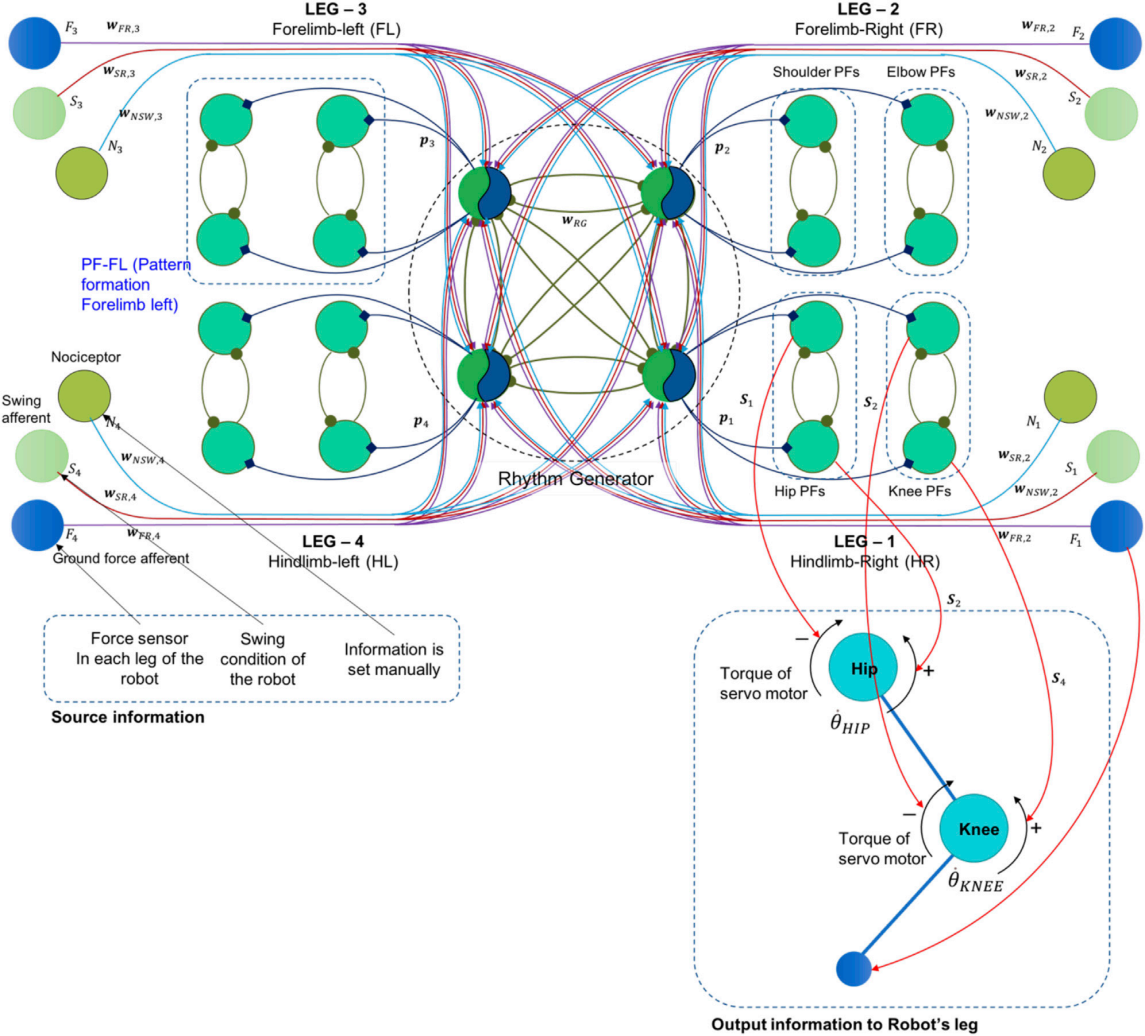
The single-rhythm CPG model with a two-layered CPG. The rhythm generator neurons received feedback signals from a force sensor, a pain receptor, and a swing sensor in each leg.

We designed a single-model CPG in which each RG neuron represents the movement pattern of one leg, and each pattern formation (PF) neuron represents the activation of one muscle. Since we use four muscles in one leg (flexor and extensor muscles of hip and knee joint), each limb structure in the CPG network comprises one RG neuron and four PF neurons. Our model uses two CPGs, one for the forelimbs, and one for the hindlimbs. The overall CPG design can be seen in Figure 10. We extend the CPG model from our previous model published in (Saputra et al., 2020b). We used the Matsuoka neural-oscillator model to generate a dynamic signal. The inner state of the RG neuron can be seen in this following equation:

$$\tau \frac{d}{dt}x_{i}=\left(v_{i}-x_{i}-\overset{n}{\underset{j=1}{\sum }}w_{\left(RG,ij\right)}y_{j}+\alpha_{i}-bv_{i}\right)\left(\tau_{f}S_{STIM}\right)$$ (1)

$$T\frac{d}{dt}v_{i}=\left(y_{i}-v_{i}\right)\left(\tau_{f}S_{STIM}\right)$$ (2)

$$\alpha_{i}=\alpha_{i,0}+\overset{n}{\underset{j=1}{\sum }}w_{\left(FR,ij\right)}F_{i}N_{j}-\overset{n}{\underset{j=1}{\sum }}(G_{STIM,A}\left(S_{i}\right)(w_{\left(SR,ij\right)}N_{j})-\overset{n}{\underset{j=1}{\sum }}\left(G_{STIM,B}\left(S_{i}\right)w_{\left(NS,ij\right)}N_{j}\right)$$ (3)

$$G_{STIM,A}=3/\left(1\;+\exp\left(-8\;\;S_{STIM}\;+\;15\right)\right)$$ (4)

$$G_{STIM,B}=2\exp\left(\log\left(0.5\right)\;{\left(2\;S_{STIM}-4\right)}^{2}\right)$$ (5)

The signal from RG neurons will be transmitted to the PF neurons. PF neuron will generate spike activation for swinging action in certain leg. The spike signal of PF neurons (*P*_*i*_) is calculated in the Eq. 6, where the references signal (*h*_*i*_^*ref*^) is calculated in Eq. 7, *R* is subtraction constant parameter, *γ* ^*ref*^ is discount rate of *h*_*i*_^*ref*^, and *q* is the spike threshold. We also provide a source code of the CPG model in the attached link of Supplementary Material S3.

$$p_{i}\left(t\right)=\{\begin{array}{cc} 1 & if\;\left(y_{i}+h_{i}^{ref}\left(t\right)\right)>q\\ 0 & Otherwise \end{array}\;$$ (6)

$$h_{i}^{ref}\left(t\right)=\begin{array}{cc} \gamma^{ref}\Delta h_{i}^{ref}\left(t-1\right)-R & if\;(P_{i}\left(t-1\right)=1)\\ \gamma^{ref}\Delta h_{i}^{ref}\left(t-1\right) & Otherwise \end{array}$$ (7)

$$PF_{i,k}\left(t\right)=e^{\left(c_{1}\left(\frac{\left|P_{i,k}\left(t\right)-\mu \right|}{\mu \Delta w}\right)\right)},\;P_{i,k}\left(t\right)=P_{i,k}\left(t\right)+p_{i}\left(t\right)$$ (8)

In the process, RG neurons have a rhythmic pattern signal and generate the spike signal to the PF neurons. The parameter *PF*_*i,k*_(*t*)in Eq. 8 is the signal generated by *k*th of PF neuron in *i*th leg. It will activate the muscle stimulation explained in the previous research (Saputra et al., 2020b)(Saputra et al., 2020a). The output of the muscle stimulation (*S*_*i*_) will be converted to the direction the torque of servo actuator in the robot’s leg. The connection information can be seen in the Figure 10. Torque of one servo motor is driven by two muscle stimulation for different direction, flexor muscle stimulation is for CW direction and extensor stimulation is for CCW direction. Regarding to the Figure 10, the total torque and the servo angular velocity are approached by Eqs. 9 Eqs. 10, where (*r*) is the attachment length of muscle assumption, defined as 0.03 m.

$$\tau_{HIP}\left(t\right)=rS_{1}\left(t\right)+rS_{2}\left(t\right)\;$$ (9)

$${\dot{\theta }}_{HIP}\left(t\right)={\dot{\theta }}_{HIP}\left(t-1\right)+\left(S_{1}\left(t\right)-rS_{2}\left(t\right)\right)/r\;$$ (10)

## Robot Implementation

In order to test and demonstrate the robot’s capabilities, we had the robot move across natural terrain, walk with a leg malfunction, avoid a sudden obstacle while walking, and climb on a vertical ladder. The optimization process of CPG model and muscle activation function can be seen in our previous papers (Saputra et al., 2020b)(Saputra et al., 2020a).

### Moving on Natural Terrain

We trialed the robot on natural terrain (grassed soil with varying slope) and flat terrain (a carpeted floor). The robot’s gait pattern was controlled using the proposed neural-network locomotion generator. The optimized parameter of CPG model as pattern generation can be seen in Table 6.

**TABLE 6:** Optimized parameter of CPG.

τ	*T*	*b*	*τ*_*f*_	Time step (s)	α_*i,0*_	*γ* ^*ref*^	*Q*	*R*
1.0	12.0	1.5	3	0.01	1.0	0.98	0.5	30
***w*** **_(*RG*,*ij*)_**	***w*** **_(*RG*,*i*1)_**	***w*** **_(*RG*,*i*2)_**	***w*** **_(*RG*,*i*3)_**	***w*** **_(*RG*,*i*4)_**	***w*** **_(*FR*,*ij*)_**	***w*** **_(*FR*,*i*1)_**	***w*** **_(*FR*,*i*2)_**	***w*** **_(*FR*,*i*3)_**	***w*** **_(*FR*,*i*4)_**
***w***_(*RG*,1*j*)_	0.00	2.431	1.32	2.431	***w***_(*FR*,1*j*)_	0.00	1.002	0.960	1.023
***w***_(*RG*,2*j*)_	1.32	0.00	2.431	2.431	***w***_(*FR*,2*j*)_	0.960	0.00	1.023	1.002
***w***_(*RG*,3*j*)_	2.431	2.431	0.00	1.32	***w***_(*FR*,3*j*)_	1.002	1.023	0.00	0.960
***w***_(*RG*,4*j*)_	2.431	1.23	2.431	0.00	***w***_(*FR*,4*j*)_	1.023	0.960	1.002	0.00
***w*** **_(*SR*,*ij*)_**	***w*** **_(*SR*,*i*1)_**	***w*** **_(*SR*,*i*2)_**	***w*** **_(*SR*,*i*3)_**	***w*** **_(*SR*,*i*4)_**	***w***_(*NS*,*ij*)_	***w***_(*NS*,*i*1)_	***w***_(*NS*,*i*2)_	***w***_(*NS*,*i*3)_	***w***_(*NS*,*i*4)_
***w***_(*SR*,1*j*)_	0.00	0.00	2.059	0.00	***w***_(*NS*,1*i*)_	0.00	0.001	0.00	0.01
***w***_(*SR*,2*j*)_	0.02	0.00	0.00	0.00	***w***_(*NS*,2*j*)_	1.875	0.00	0.00	0.00
***w***_(*SR*,3*j*)_	0.00	0.00	0.00	0.02	***w***_(*NS*,3*j*)_	0.00	0.00	0.00	1.94
***w***_(*SR*,4*j*)_	0.00	1.987	0.00	0.00	***w***_(*NS*,4*j*)_	0.00	0.00	0.001	0.00

**FIGURE 11:**
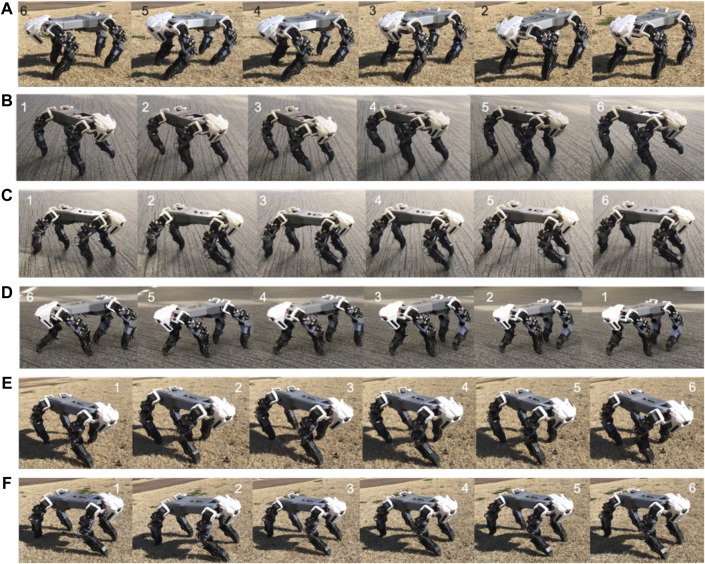
The robot’s dynamic gait pattern **(A)** on natural terrain **(B)** on flat terrain. **(C)** Dynamic gait pattern on flat terrain with injured forelimb. **(D)** On flat terrain with injured hindlimb. **(E)** On natural terrain with injured forelimb. **(F)** On natural terrain with injured hindlimb. The video of robot’s performance can be seen in the link of Video 2.

**FIGURE 12:**
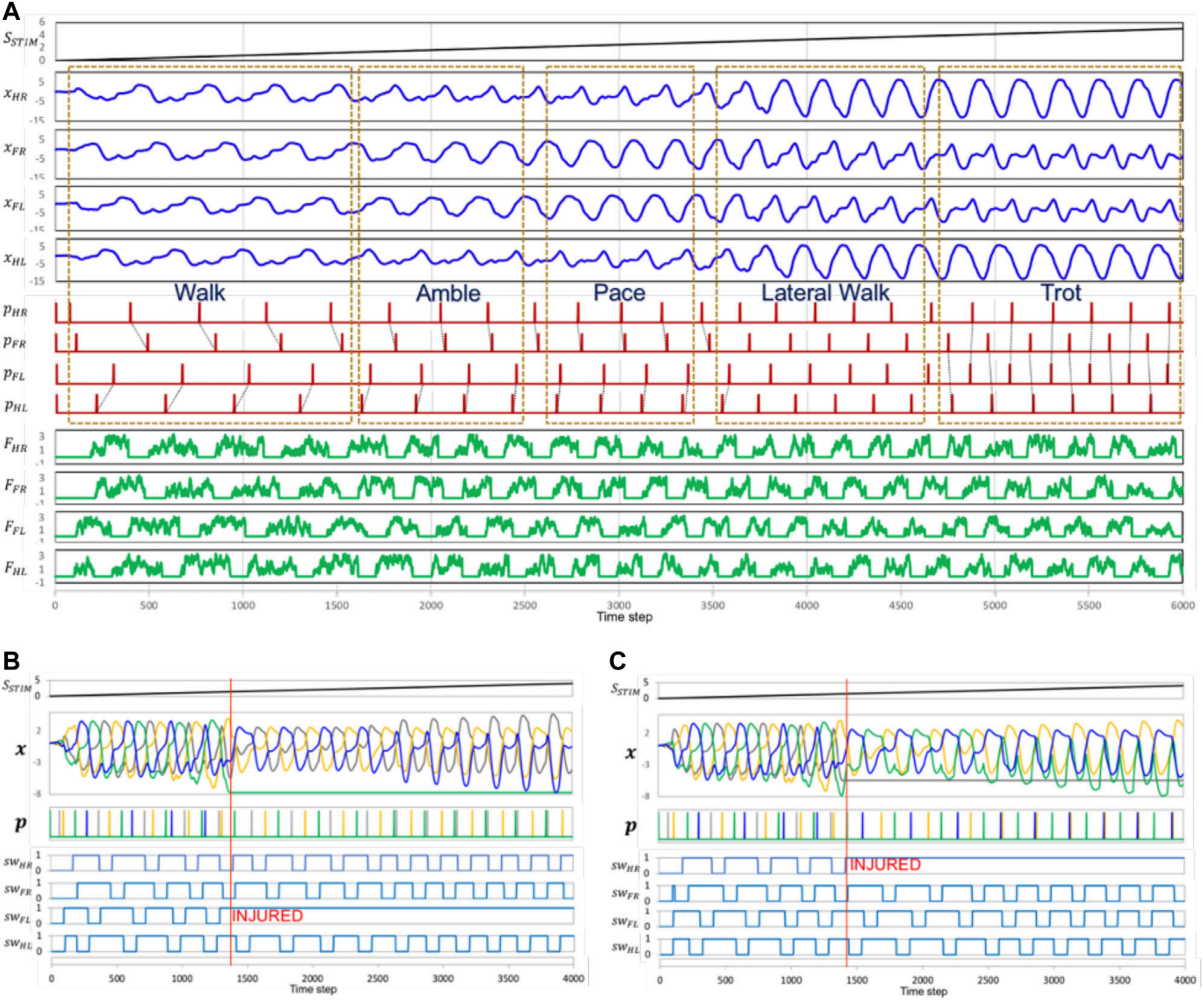
**(A)** The locomotion model can generate dynamic gait pattern by giving different speed stimulation. It shows there are five different known gait patterns from slow speed to high speed. The CPG model can generate a dynamic gait pattern through differing speed stimulation ***S***_***STIM***_. This increases the frequency of the CPG outputs ***x*** of five different known gait patterns from slow speed to high speed. Parameter *F*_*HR*_ *, F*_*FR*_ *, F*_*HL*_ *, F*_*FL*_ shows the ground reaction force for every limb. **(B)** The generated gait patterns in malfunction conditions and the speed stimulation responses. The signal pattern *p* is changing to respond to the absence of CPG signals. The time phase decreases after a leg is injured. The malfunction of the right forelimb (*N*_*FL*_ = 1) is at time step 1380. During injury, the model tends to generate a pattern with the same phase difference at a lower speed. At high speeds, left and right hindlimbs feature the same phase. **(C)** malfunction of the left hindlimb at time step 1400. In this condition, the left and right forelimbs feature the same phase at a higher speed.

Sample snapshots of the robot’s performance can be seen in Figures 11A,B. We set *S*_*STIM*_ from zero and gradually increase along the value of time step (*S*_*STIM*_ = time step/1200). The result can be seen in Figure 12A. The CPG model can generate dynamic gait pattern. The robot can produce dynamic gait patterns to walk, amble, pace and trot successfully across both terrains.

### Moving With a Leg Malfunction

We tested the robot in two conditions: 1) with an injured forelimb, and 2) with an injured hindlimb. Both tests began with the robot in normal gait. After a few seconds, we set one of the legs to its ‘injured’ state. In this case the locomotion model cannot generate signal to the injured leg. However, the torque force of the leg is still active. In both tests, the robot responded by appropriately transitioning its without falling down. These tests were conducted on both artificial and natural terrain. Snapshots of the robot’s performance can be seen in Figures 11C–F. The corresponding video is can be seen in the link of Video 2. Furthermore, the movement transition when leg got injured can be analyzed in Figures 12B,C.

### Avoiding a Sudden Obstacle While Moving

**FIGURE 13:**
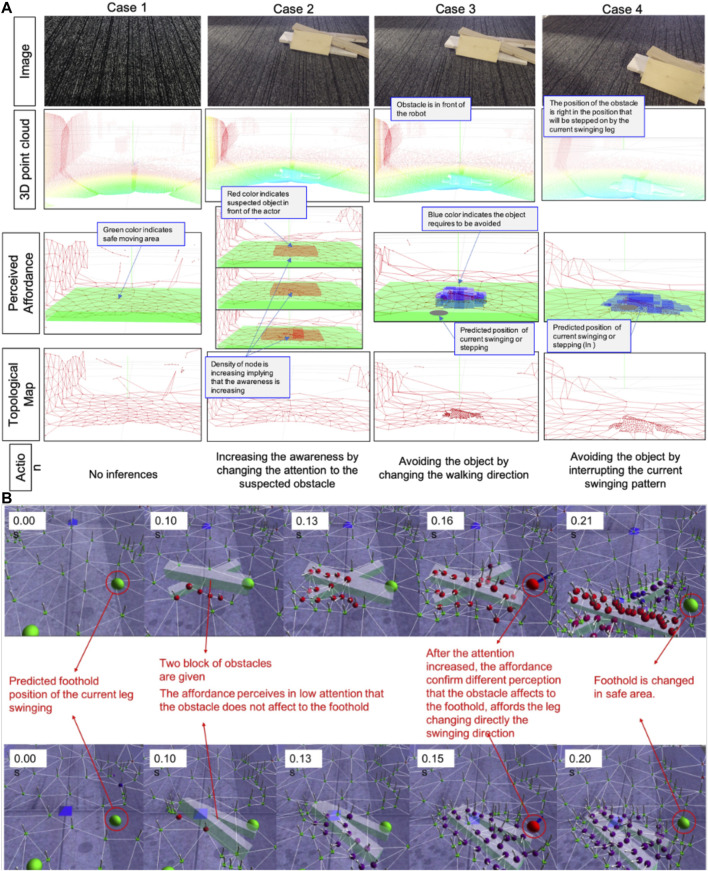
**(A)** The result of different condition perception in different levels of data (3D point-cloud being the raw data from the depth sensors, topological map structure represent the attention model, perceived affordance, and generated action) **(B)** The snapshots show the integration between affordance and attention in computer simulation.

In this trial, we set the robot to travel straight ahead. Once it was moving, we suddenly put a few small pieces of woods in front of the robot’s front leg. This experiment tested how effectively the locomotion generator could produce short-term adaptations in response to external sensory information. The affordance process perceived the object before the robot took any action. The four columns in Figure 13A show affordance perception and adaptation in progress. An increase in map density (case 3) corresponds to the obstacle’s location. The robot performance avoiding sudden obstacle dropped into its path can be seen in the link of Video 3.

In order to show the integration of affordance and attention in robot locomotion, we first analyze the attention and affordance result in simulation, as shown in this link Video 6 and Figure 13B. Simulation proved that the degree of attention may affect the accuracy of affordance detection. The topological structure (nodes and edges) represent the attentional model. The green ball represents the predicted foothold position for the current swinging movement. We suddenly put an obstacle around that intended foothold position 0.1 S after the leg starts swinging. A few nodes appear with non-homogeneous normal vectors (red color's nodes), meaning that the affordance detector has perceived some sudden obstacle with low accuracy. In this condition, the affordance system asks the attention process to focus on the obstacle. Then, the red-colored nodes promptly generate new nodes. After 0.11 S, the number of nodes has greatly increased around the obstacle, showing that the affordance detector has perceived the obstacle with high accuracy. The robot is then directed to change its swing to a safe area (green nodes).

### Climbing on Vertical Ladder

**FIGURE 14:**
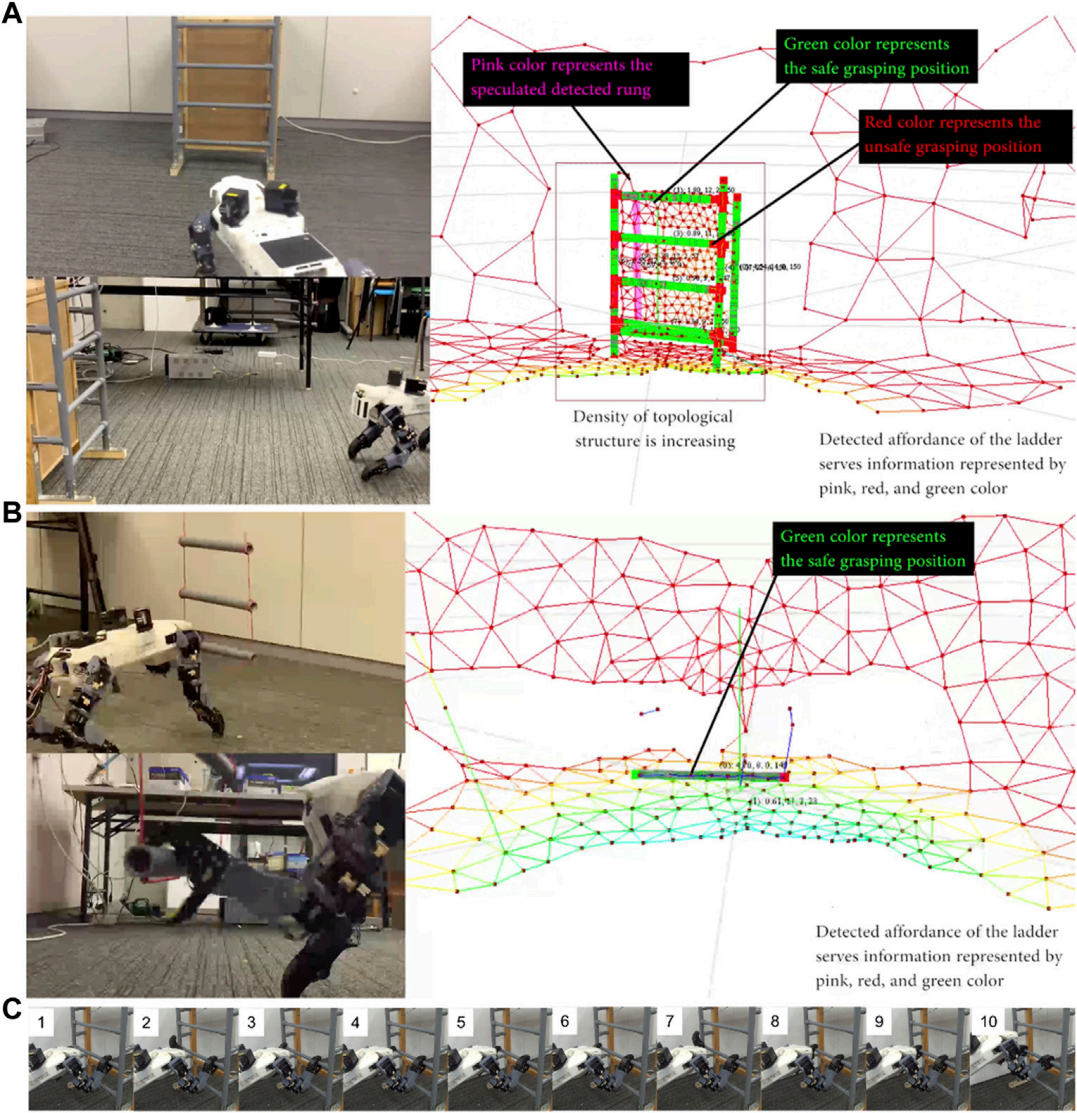
**(A)** The robot detects the ladder’s affordances while approaching it. **(B)** Robot detects graspability of a swinging ladder and grasps a rung of the moving ladder. **(C)** The snapshot video of the robot performance detailed in the link of Video 5. The robot approaches and climbs a ladder, then transitions back to a horizontal posture to stand on the tabletop (frames 9 – 10).

Before setting a climbing task, we tested the robot’s ability to detect and interpret a vertical ladder detection using an inlier–outlier method. Affordance detection, in this case, is directed toward finding feasibly graspable locations. The detail affordance detection is explained in the (Saputra et al., 2019b). Figure 14A shows the robot detecting and tracking the ladder structure in real time, identifying which parts it can safely grasp. The detail video can be seen in the link of Video 4.

The robot’s next task is to walk to the ladder and climb up it onto a higher floor, all without handrail support. This task entails transitions from horizontal motion to vertical motion, and then from vertical motion to horizontal motion. To tackle this problem, we propose an additional behavior generation model using independent stepping and pose control in the robot. Posture, safe movement areas, possible touch points, graspability, and target movement all need to be determined from the robot’s sensors. As noted in our earlier research, four kinds of behavior are required: approaching, body–placing, stepping, and grasping (Saputra et al., 2019c). The proposed model was first optimized through simulation. The robot, in turn, successfully moved from the lower level to the upper level, negotiating the ladder between them (Figure 14C). The video of the robot performance climbing the vertical ladder can be seen in the link of Video 5.

## Conclusion and Future Plans

We developed a robot inspired by domestic feline morphology. The main contribution of the proposed robot is finding some benefit of biological morphology for robotics to tackle unsolved terrain. We imitate the morphology of the Cat animal in the robot structure and the paw mechanism. In the sensory system, we design the novel structure of 3D point cloud sensors for improving the efficiency. Then, the robot is built to show some novel bio-inspired model. The robot responds to both internal and external sensory information, processing the sensory input through several bio-inspired novel capabilities that enable the robot’s motion through complicated terrains. The robot, though built on a low-cost budget (estimated as 12.000 USD), has been successfully trialed in several environmental conditions. The locomotion model of the robot can generate a dynamic gait pattern by stimulated only one single speed parameter. There are five patterns generated in the robot performance, walk, amble, pace, symmetrical walk, and trot gait. There are, however, still some practical problems still to be solved. Our continuing research will focus on these three areas:

ߦ Improving stability: We will improve the robot’s use of its inertial sensor data in manipulating the current stability model.
ߦ Soften footfall: the robot’s step is currently heavy. We will add a damper mechanism to soften footfall, inspired by feline leg structure.
ߦ Improving durability: the robot needs to run for longer. This may be achieved by increasing battery capacity and body efficiency, for example by decreasing the robot’s weight.
ߦ Advanced terrain handling: further experimentation is required to develop the robot’s performance in more complex environments. We will design and build an artificial ruin in which to develop and test the robot.

## Link to Online Video

Video 1: https://youtu.be/9MEojC5SjdA Shows 3D point clouds data generated by Quad ToF sensor, Comparison of the proposed dynamic density topological generator with other model, The proposed model can specified the density in the obstacle area automatically.
Video 2: https://youtu.be/4NeW1u3OfFo Shows the robot performance in natural and rough terrain, dynamic gait transition in different speed, and robot’s performance during malfunction condition.
Video 3: https://youtu.be/TYACHd9G88E Shows the Robot Performance Avoiding Sudden Obstacle While Moving
Video 4: https://youtu.be/4sZH1vKzNp0 Shows the performance of real time vertical ladder affordance detection while approaching the ladder and performance of moving ladder affordance detection.
Video 5: https://youtu.be/Y_lmzQf-3Lk Shows the novel capabilities of the robot moving through the vertical ladder without handrail support.
Video 6: https://youtu.be/hfL9vE847Es Shows the integration between affordance and attention in computer simulation.

## Data Availability

The original contributions presented in the study are included in the article/Supplementary Material, further inquiries can be directed to the corresponding author.
